# Study on the technology of enhancing permeability by deep hole presplitting blasting in Sanyuan coal mine

**DOI:** 10.1038/s41598-021-98922-9

**Published:** 2021-10-13

**Authors:** Dan Zhao, Mingyu Wang, Xinhao Gao

**Affiliations:** 1 College of Safety Science and Engineering, Liaoning Technical University, Fuxin, 123000 China; 2 Power China Huadong Engineering Corporation Limited, Hangzhou, 311122 Zhejiang China; 3 Key Laboratory of Mine Power Disaster and Prevention of Ministry of Education, Huludao, 125105 Liaoning China

**Keywords:** Environmental chemistry, Environmental chemistry, Chemical engineering

## Abstract

To reduce gas disasters in low permeability and high gas coal seams and improve gas predrainage efficiency, conventional deep hole presplitting blasting permeability increasing technology was refined and perfected. The damage degree of coal and rock blasting was quantitatively evaluated by using the value range of the damage variable *D*. According to the actual field test parameters of coal seam #3 in the Sanyuan coal mine, *D*_lim_ = 0.81 ~ 1.0 was the coal rock crushing area, *D*_lim_ = 0.19 ~ 0.81 was the coal rock crack area, and *D*_lim_ = 0 ~ 0.19 was the coal rock disturbance area. The blasting models under different blasting parameters were established by ANSYS/LS-DYNA software. The influence radius of single-hole blasting was 3.1 m, the hole diameter of double-hole blasting was 113 mm, the hole spacing was 5.5 m, and the delayed blasting time was 25 ms. According to the numerical simulation results, the determined parameters were tested on the working face of the 1312 transportation roadway in coal seam #3 of the Sanyuan coal mine. The results show that after blasting, the permeability of the original coal seam was increased by more than 30 times, the gas concentration was increased by 2.16 times, and the single hole purity and mixing volume were increased by 4.73 and 4.27 times, respectively. The positive effects of deep hole presplitting blasting permeability enhancement technology on the pressure relief and permeability enhancement of a low pressure and high gas coal seam were determined.

## Introduction

Coal seam gas predrainage technology is considered to be one of the most effective measures to prevent coal mine gas disasters^1–6^. However, with increasing mining depth, the gas pressure of the coal seam increases and the permeability decreases, which seriously affects the gas predrainage effect^7–9^. In engineering practice, the deep hole presplitting blasting permeability increasing technology can produce multiple cracks in the coal body of a low permeability coal seam, forming an underground fracture network channel, which can greatly improve the gas drainage efficiency and reduce or even eliminate the hidden dangers in the gas mining process^10–13^.

Research on deep hole presplitting blasting in high gas coal seams began in the last century. Badal^14^ established an indoor experimental model for comparative tests and concluded that the combined effect of the explosion shock wave and explosion gas produced by the explosion is an important cause of coal and rock fragmentation. Zhang et al.^15^ adopted advanced presplitting blasting technology to weaken the coal body and enhance the blasting effect at the same time. Paine et al.^16^ established the corresponding mathematical model by taking the change in gas generated by blasting holes and the change in ground stress before and after blasting as variables and summarized the corresponding expression and solution method. Roy et al.^17^ carried out field experimental research on blasting drilling data parameters, explosion parameters, and gas risk by using new blasting technology. Singh et al.^18^ studied the technology of controlled blasting to increase the permeability of a partially collapsed coal face caused by deep hole presplitting. Zhang^19^ conducted blasting tests in underground mines and found that deep hole controlled presplitting blasting can develop and extend the fissures in the coal body to form a fissure network, and at the same time, it can also improve the gas drainage of low permeability and high gas coal seams. Chen et al.^20^ proposed deep hole presplitting blasting for weakening thick hard roofs to mitigate strong strata behaviours. Aliabadian and Sharafisafa^21^ investigated the effect of presplitting on the generation of a smooth wall in a rock domain under the blasting process in a continuum rock mass. Chen et al.^22^ drew the following conclusion through experimental research: when the blasting control hole is in the blasting fracture zone of the goaf, the development direction of the crack can be effectively controlled, which can not only meet the requirements of blasting but also improve the gas drainage efficiency. With the rapid development of computer technology, numerical simulation has become an important means to study the theory and technology of rock blasting. Valiappan et al.^23^ analysed and discussed the detailed situation of rock blasting by using finite element calculations. Chu and Yang^24^ used the relevant knowledge of mechanics to analyse the loss fracture criterion of coal mass after blasting. The results show that the stress wave can not only help the extension of primary fractures but also accelerate the generation of a small number of new cracks, which plays a role in pressure relief for enhancing the permeability of the coal seam. Xu et al.^25^ analysed various factors that may affect the permeability enhancement effect of presplitting blasting by means of finite element software simulation, which has practical significance for the field application of presplitting blasting. Kumar et al.^26^ performed a comparative analysis on the role of deep hole presplitting blasting technology in the rock blasting process through field measurement analysis results. Gao et al.^27^, through numerical simulation of the actual situation in the field, concluded that the large number of cracks in the hard roof rock mass after deep hole blasting not only released the roof pressure but also improved the protection effect of gob-side roadway retaining.

The abovementioned scholars have performed some research on presplitting blasting technology^28–31^. However, the existing theoretical knowledge of controlled blasting is based on rock masses as the analysis object of experimental research. At present, coal and gas in mines are in the same space, and they complement each other as a whole. If we do not consider the existence of gas in the coal seam and only study the coal and rock mass as a single solid medium, some errors will exist compared to the actual situation. Therefore, in view of the technical problem of difficult gas drainage in the 1312 working face of coal seam #3 in the Sanyuan coal mine of China, this paper proposes a new technology of deep hole presplitting blasting to increase permeability. The blasting scheme is optimized by numerical simulation and practical operation. The paper has a certain reference significance for the gas control of similar coal mines in the same area.

## Mathematical model and boundary conditions

### Mathematical model

Solid basic theory and a variety of algorithms are the foundation of the powerful function of LS-DYNA. The following is a brief introduction to its basic theory^32–34^.

1. Momentum conservation equation

1 $$\frac{{\partial \sigma_{ij} }}{{\partial x_{j} }} + \rho f_{i} = \rho x_{i}$$

where *σ*_*ij*_ is the stress vector, *x* is the acceleration of the particle (m·(s^2^)^−1^), *ρ* is the gas density (kg·(m^3^)^−1^), and *f*_*i*_ is the mass volume force. $x_{i}$ is the equation of motion of the coordinate $x_{i}$(1,2,3) at any time.

The displacement boundary conditions shall meet the following requirements:

2 $$x_{i} = \overline{x}_{i}$$

where $\overline{x}$ is the displacement function on the displacement boundary.

The stress boundary conditions shall meet the following requirements:

3 $$\sum\limits_{j = 1}^{3} {\sigma_{ij} n_{i} } = \overline{T}_{i}$$

where *n*_*i*_ is the configuration boundary and $\overline{T}_{i}$ is the force load on the boundary.

(2) Conservation equation of mass

4 $$\rho V = \rho_{0}$$

where *ρ* is the gas density (kg·(m^3^)^-1^), *ρ*_0_ is the initial density (kg·(m^3^)^-1^), and *V* is the relative volume.

(3) Energy conservation equation

5 $$E^{ * } = V_{i} S_{ij} \varepsilon_{ij} - (P_{g} + q^{ * } )V$$

where *E* is the energy, *V* is the configuration volume (m^3^), *S*_*ij*_ is the deviator stress (MPa), *ε*_*ij*_ is the strain rate tensor, *P*_*g*_ is the pressure (MPa), q^*^ is the volume viscous resistance (Pa), and $V_{i}$ is the initial velocity of the point (m/s).

### Selection of material model and determination of parameters

1. Material selection of the coal model

To more accurately express the large deformation failure mechanism of coal and rock under dynamic loading, elastic–plastic dynamic material is used for the coal material. Detailed material parameters are shown in Table 1.

(2) Material selection of the air model

**Table 1:** Rock material parameters.

Name	Coal rock density	Elastic modulus	Poisson’s ratio	Tensile strength	Compressive strength	Cohesion
Value	1.42 g/cm^−3^	0.855 GPa	0.323	1.909 MPa	10.848 MPa	1.142 MPa

The Unit *MAT_NULL air material model provided in the program is selected in this paper, and the equation of state can be expressed as Eq. (6). The *EOS_LINEAR_POLYNOMIAL keyword in the system is used to define the equation of state.

6 $$P = C_{0} + C_{1} \mu + C_{2} \mu^{2} + C_{3} \mu^{3} + (C_{4} + C_{5} \mu + C_{6} \mu^{2} )E_{2}$$

where *C*_1_, *C*_2_, *C*_3_, *C*_4_, *C*_5_, and *C*_6_ are the material parameters and *E*_2_ is the internal energy per unit volume (GPa). Detailed material parameters are shown in Table 2.

(3) Damage variable *D*

**Table 2:** Air material parameters.

Density	*C*_0_	*C*_1_	*C*_2_	*C*_3_	*C*_4_	*C*_5_	*C*_6_	*E*_2_
1.29E^−5^	0	0	0	0	0.4	0.4	0	0.025
Unit	g/cm	g/cm	g/cm	g/cm	g/cm	g/cm	g/cm	GPa

The damage equation is as follows:

7 $$E = (1 - D)E_{0}$$

where *D* is the damage variable, *E* is the elastic modulus of the damaged element, and *E*_0_ is the elastic modulus of the lossless element.

When the shear stress reaches the Mohr–Coulomb criterion damage threshold,

8 $$F = \sigma_{2} - \sigma_{3} \frac{1 + \sin \psi }{{1 - \sin \psi }} \ge f_{c}$$

where *F* is the shear stress (MPa), σ_2_ and σ_3_ are the static and dynamic failure strengths (MPa), $\psi$ is the angle of internal friction (°), and *f*_*c*_ is the compressive strength (MPa).

The damage variable *D* is expressed as:

9 $$D = \left\{ \begin{gathered} 0{\kern 1pt} {\kern 1pt} {\kern 1pt} {\kern 1pt} {\kern 1pt} {\kern 1pt} {\kern 1pt} {\kern 1pt} {\kern 1pt} {\kern 1pt} {\kern 1pt} {\kern 1pt} {\kern 1pt} {\kern 1pt} {\kern 1pt} {\kern 1pt} {\kern 1pt} {\kern 1pt} {\kern 1pt} {\kern 1pt} {\kern 1pt} {\kern 1pt} {\kern 1pt} {\kern 1pt} {\kern 1pt} {\kern 1pt} {\kern 1pt} {\kern 1pt} {\kern 1pt} {\kern 1pt} {\kern 1pt} {\kern 1pt} {\kern 1pt} {\kern 1pt} {\kern 1pt} {\kern 1pt} {\kern 1pt} {\kern 1pt} {\kern 1pt} {\kern 1pt} {\kern 1pt} {\kern 1pt} {\kern 1pt} {\kern 1pt} {\kern 1pt} {\kern 1pt} \varepsilon < \varepsilon_{c0} \hfill \\ 1 - \frac{{f_{cr} }}{{E_{0} \varepsilon }}{\kern 1pt} {\kern 1pt} {\kern 1pt} {\kern 1pt} {\kern 1pt} {\kern 1pt} {\kern 1pt} {\kern 1pt} {\kern 1pt} {\kern 1pt} {\kern 1pt} {\kern 1pt} {\kern 1pt} {\kern 1pt} {\kern 1pt} {\kern 1pt} {\kern 1pt} \varepsilon_{c0} \le \varepsilon_{r} {\kern 1pt} {\kern 1pt} {\kern 1pt} \hfill \\ \end{gathered} \right.{\kern 1pt} {\kern 1pt} {\kern 1pt} {\kern 1pt} {\kern 1pt} {\kern 1pt} {\kern 1pt} {\kern 1pt} {\kern 1pt} {\kern 1pt} {\kern 1pt} {\kern 1pt} {\kern 1pt} {\kern 1pt} {\kern 1pt}$$

where *f*_*cr*_ is the compressive residual strength (MPa), *ε*_*c*0_ is the maximum compressive strain, and *ε*_*r*_ is the residual strain.

The permeability coefficient of the corresponding unit is expressed as follows:

10 $$\lambda = \left\{ \begin{gathered} \lambda_{0} {\text{e}}{\kern 1pt} {\kern 1pt} {\kern 1pt}^{{ - \beta_{1} (\sigma_{2} - \alpha_{1} p)}} {\kern 1pt} {\kern 1pt} {\kern 1pt} {\kern 1pt} {\kern 1pt} {\kern 1pt} {\kern 1pt} {\kern 1pt} {\kern 1pt} {\kern 1pt} {\kern 1pt} {\kern 1pt} {\kern 1pt} {\kern 1pt} {\kern 1pt} {\kern 1pt} {\kern 1pt} {\kern 1pt} {\kern 1pt} {\kern 1pt} {\kern 1pt} {\kern 1pt} {\kern 1pt} {\kern 1pt} {\kern 1pt} {\kern 1pt} D = 0 \hfill \\ \xi \lambda_{0} {\text{e}}{\kern 1pt} {\kern 1pt} {\kern 1pt}^{{ - \beta_{1} (\sigma_{2} - \alpha_{1} p)}} {\kern 1pt} {\kern 1pt} {\kern 1pt} {\kern 1pt} {\kern 1pt} {\kern 1pt} {\kern 1pt} {\kern 1pt} {\kern 1pt} {\kern 1pt} {\kern 1pt} {\kern 1pt} {\kern 1pt} {\kern 1pt} {\kern 1pt} {\kern 1pt} {\kern 1pt} {\kern 1pt} {\kern 1pt} {\kern 1pt} D > 0{\kern 1pt} {\kern 1pt} {\kern 1pt} \hfill \\ \end{gathered} \right.{\kern 1pt} {\kern 1pt} {\kern 1pt} {\kern 1pt} {\kern 1pt} {\kern 1pt} {\kern 1pt} {\kern 1pt} {\kern 1pt} {\kern 1pt} {\kern 1pt} {\kern 1pt} {\kern 1pt} {\kern 1pt} {\kern 1pt}$$

where *λ*_0_ is the air permeability coefficient (m^2^·(MPa^2^·d)^−1^), *ξ* is the increasing factor of the permeability coefficient (m^2^·(MPa^2^·d)^−1^), *α*_1_ is the gas pressure coefficient, and *β*_1_ is the stress influence coefficient.

When the element reaches the damage threshold of tensile strength *f*_*t*_,

11 $$\sigma_{3} \le - f_{t}$$

The damage variable *D* is expressed as follows:

12 $$D = \left\{ {\begin{array}{*{20}c} 0 & {\varepsilon \le \varepsilon_{t0} } \\ {1 - \frac{{f_{tr} }}\;{{E_{0} \varepsilon }}} & {\varepsilon_{t0} \le \varepsilon \le \varepsilon_{tu} } \\ 1 & {\varepsilon \ge \varepsilon_{tu} } \\ \end{array} } \right.$$

where *f*_*tr*_ is the tensile residual strength (MPa), *ε*_*r*_ is the residual strain, and *ε*_*t*0_ is the maximum tensile strain.

The corresponding unit permeability coefficient is described as follows:

13 $$\lambda = \left\{ {\begin{array}{*{20}c} {\lambda_{0} e^{{ - \beta_{1} \left( {\sigma_{3} - ap} \right)}} } & {D = 0} \\ {\xi \lambda_{0} e^{{ - \beta_{1} \left( {\sigma_{3} - ap} \right)}} } & {0 < D < } \\ {\xi^{\prime } \lambda_{0} e^{{ - \beta_{1} \left( {\sigma_{3} - ap} \right)}} } & {D = 1} \\ \end{array} } \right.1$$

where *ξ* is the increasing factor of the permeability coefficient (m^2^·(MPa^2^·d)^−1^).

The coal rock failure criterion is expressed as:

14 $$\left\{ \begin{gathered} - \left( {\sigma_{\theta }^{ * } } \right)_{\max } > \sigma_{td} = - \left( {\sigma {}_{\theta }} \right)_{\max } /\left( {1 - D_{\lim } } \right){\kern 1pt}\; (fracturezone) \hfill \\ {\kern 1pt} \left( {\sigma_{r}^{ * } } \right)_{\max } > \sigma_{cd} = \left( {\sigma {}_{r}} \right)_{\max } /\left( {1 - D_{\lim } } \right)\;(crushzone) \hfill \\ \end{gathered} \right.$$

where $\left( {\sigma_{r}^{ * } } \right)_{\max }$ is the maximum radial effective stress (MPa), $\left( {\sigma_{\theta }^{r} } \right)_{\max }$ is the maximum effective circumferential stress(MPa), $\left( {\sigma_{\theta }^{{}} } \right)_{\max }$ is the maximum nominal toroidal dynamic stress (MPa), $\left( {\sigma_{r}^{{}} } \right)_{\max }$ is the maximum nominal radial dynamic stress (MPa), $\sigma_{cd}$ is the uniaxial dynamic compressive strength of coal and rock (MPa), $\sigma_{td}$ is the uniaxial dynamic tensile strength of coal and rock (MPa), and *D*_*lim*_ is the critical damage variable. It is generally measured by the dynamic loading test results of medium and high strain rates in the laboratory.

On the basis of the existing blasting damage model, combined with the actual situation of coal seam #3 in the Sanyuan coal mine, the experimental results show that the coal rock crushing area occurs when D_lim_ = 0.81 ~ 1.0, the coal rock fracture area occurs when D_lim_ = 0.19 ~ 0.81, and the coal rock disturbance area occurs when D_lim_ = 0 ~ 0.19.

The damage variable *D* was used to characterize the damage degree of the blasting rock mass:

15 $$D = \sum {\frac{{\Delta \varepsilon_{{\text{p}}} + \Delta \mu_{p} }}{{D_{1} \left( {P^{ * } + T^{ * } } \right)^{{D_{2} }} }}}$$

where *D* is the damage variable, $\Delta \varepsilon_{p}$ is the increment of equivalent plastic strain, $\Delta \mu_{p}$ is the equivalent volumetric strain increment, *D*_*1*_ and *D*_*2*_ are the material parameters, $T^{ * }$ is the maximum tensile hydrostatic pressure (MPa), and $P^{ * }$ is the standardized pressure (MPa).

Obviously, the greater the damage factor *D* value is, the higher the damage degree of coal rock: when *D* = 1, the coal rock is in the state of complete crushing; when *D* is between 0 and 1, the coal rock is in the state of damage, and the coal body exhibits cracks with different development degrees. Therefore, this paper uses the value range of *D* to quantitatively evaluate the damage degree of coal and rock blasting. Usually, the fracture zone near the borehole after coal rock blasting is caused by the compression of the rock mass greater than its own compressive strength. The crack zone is the result of the rock mass being destroyed by an external tensile force greater than its own tensile strength.

### Boundary condition

When LS-DYNA is used to simulate deep hole presplitting blasting, the following boundary conditions are applied to the numerical model according to the actual situation and the relevant needs of the simulation: the upper boundary of the model will be affected by the self weight stress of the coal and rock mass. It can be expressed as follows:

16 $$q = \gamma gH$$

where *q* is the top pressure (kN·(m^2^)^−1^), *γ* is the average bulk density of the coal seam (kN·(m^2^)^−1^), *γ* is taken as 1.35 t (m^3^)^−1^, *H* is the buried depth of the coal seam (m), and *H* is taken as 900 m.

$$q = 900 \times 9.8 \times 1.35 = 11907\,{\text{kN}} \cdot ({\text{m}}^{2} )^{ - 1} = 11.907\,{\text{MPa}}$$

To optimize the simulation, the pressure *q* on the upper boundary is set as 12 MPa.

### Construction of the numerical model

**Figure 1:**
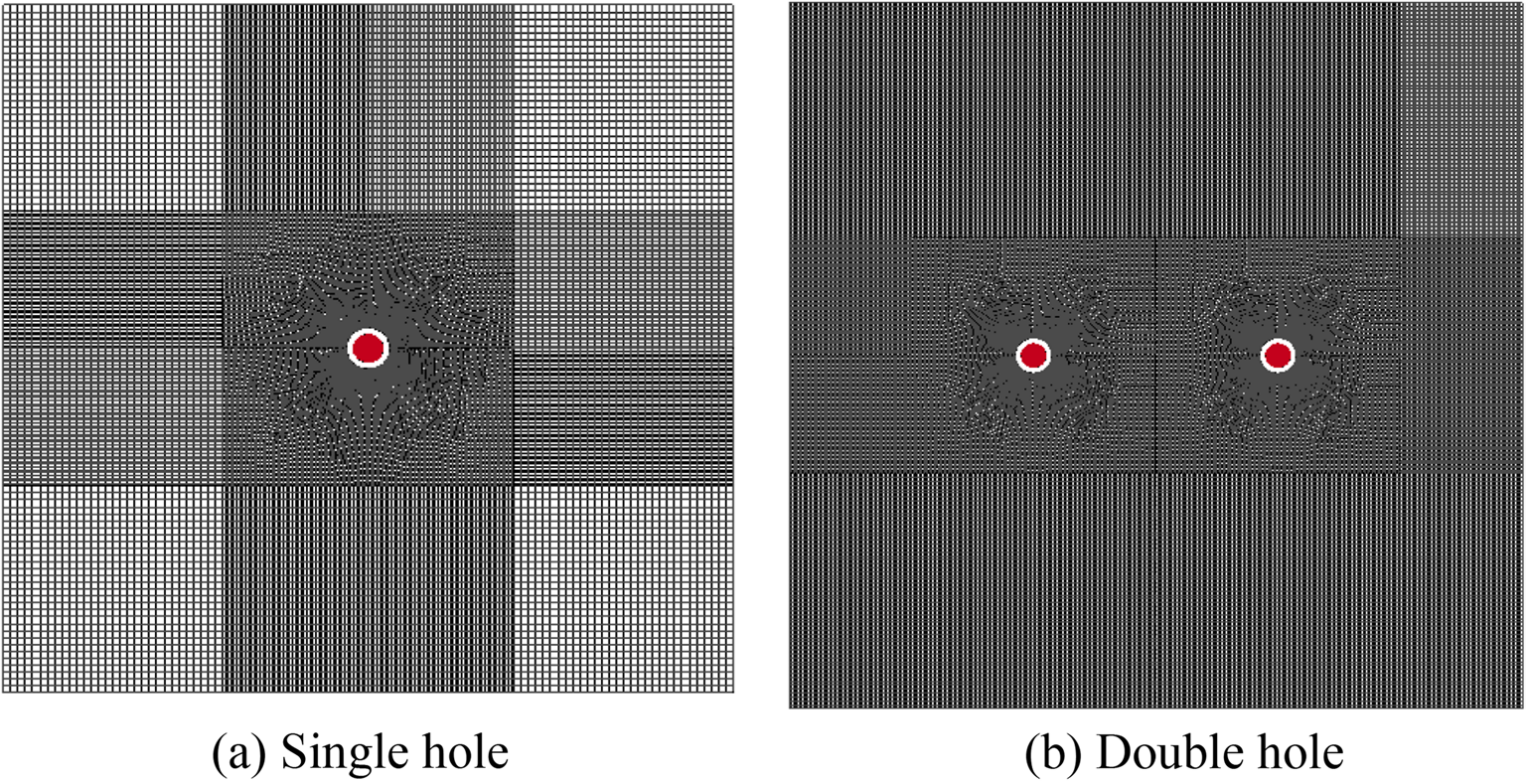
Model meshing.

The establishment of the numerical model was based on the actual situation of the 1312 working face in coal seam #3. The size of the single blasting hole numerical simulation model was 15 m × 15 m. The size of the numerical model of double blasting holes was 25 m × 25 m, and the calculation was carried out by the solid fluid coupling method. The single blasting hole was divided into 600 × 600 units, and the double blasting hole was divided into 1000 × 1000 units, as shown in Fig. 1. Horizontal constraints were added to the two ends of the model, and constraints were fixed at the bottom. Because the test site was an infinite space and the size of the numerical simulation model was limited, the problem analysis could only be carried out in a limited area. Therefore, adding non reflection boundary conditions around the model could effectively eliminate the limitation of the model boundary.

## Analysis of numerical simulation results

### Simulation analysis of single blasting hole diameter optimization

The radial uncoupled charge was used to simulate. According to the charge diameter of 50 mm used in the field test of the coal seam in the Sanyuan coal mine, numerical simulation models of single hole blasting with different apertures of 75, 94, 113 and 133 mm were established to optimize the blasting aperture.

**Figure 2:**
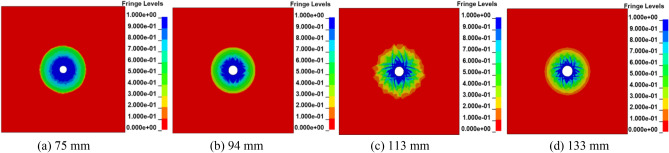
Damage distribution cloud map of coal with different pore sizes.

Figure 2 shows that the overall damage distribution of the coal and rock mass changes with the change in blasting hole diameter. According to the regional analysis of the coal near the blasting hole in the high damage state (*D* = 0.81 ~ 1), the crushing area with a 75 mm aperture is the largest, and the corresponding energy consumption is the highest. The overall fracturing effect of blasting is the worst, followed by the 94 mm aperture, and the crushingn situations of the 113 mm and 133 mm apertures are similar. The area of crushing is the smallest, the energy consumption is the least, and the range of stress wave propagation is relatively larger. With time, the intensity of the stress wave in propagation decreases gradually. When the coal and rock are in a state of incomplete damage (the *D* value is between 0.19 and 0.81), it can be seen that the fracture zone with a pore diameter of 113 mm has the best development, and its depth of influence is up to 3.10 m. The influence depths of the apertures of 75 mm, 94 mm and 133 mm are 2.48 m, 2.51 m and 2.78 m, respectively. The comprehensive consideration shows that when radial uncoupled charge is adopted, the cushioning effect generated by the 113 mm blasting hole aperture is more obvious, the energy consumption is less, the fracture density is greater, and the stress propagation distance is longer. If the distribution characteristics of the coal blasting damage area are taken as the evaluation index, it can be concluded that the effective impact radius of coal blasting is approximatrly 3.10 m.

### Simulation analysis of double blasting hole spacing optimization

In the process of deep hole presplitting blasting, when the tangential tensile stress of each point on the line of adjacent blasting holes is greater than or equal to the tensile strength of the coal and rock, the crack propagation between boreholes will form a connection near the centre of the line of two holes. To achieve the best blasting effect, it is necessary to optimize the hole spacing of multihole blasting. To test the penetration effect between boreholes, the coal damage coefficient *D* was used to characterize the stress characteristics of the medium.

**Figure 3:**
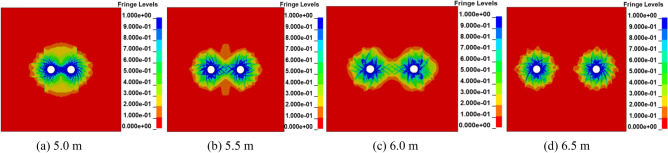
Damage distribution cloud map of different hole spacings.

The penetration of cracks between holes with different hole spacings is shown in Fig. 3. By observing the fracture morphology of damage cloud graphs with different hole spacings, it can be seen that when the adjacent hole spacing is 5.5 ~ 6.0 m, the fractures between holes can form penetration. When the adjacent hole spacing is 5.0 m, the coal damage area between the two holes (*D* = 0.81 ~ 1) is larger, resulting in a large crushing area of the coal. When the adjacent hole spacing is 6.5 m, the cracks formed between the two holes do not form an effective connection, but separate blasting fracture zones are formed. In conclusion, a hole spacing that is too large will weaken the superposition effect of the stress wave, and the adjacent holes cannot be successfully connected. A hole spacing that is too small will cause a large area of coal fragmentation. Therefore, to ensure the penetration effect of cracks and save engineering costs, 5.5 m is selected as the best spacing.

### Simulation analysis of delay time optimization of double hole millisecond blasting

This paper studies the influence of delayed blasting on the permeability enhancement effect by simulating different initiation times of blasting holes to seek the best blasting time. According to field observations and research, the commonly used differential interval time is 15 ~ 75 ms, usually 25 ~ 30 ms^35,36^. Considering the actual situation of the Sanyuan coal mine and taking the millisecond time of the high-precision detonator as the standard, the millisecond blasting time is set as 0, 17, 25 and 42 ms, and the numerical model experiment is carried out in four groups.

**Figure 4:**
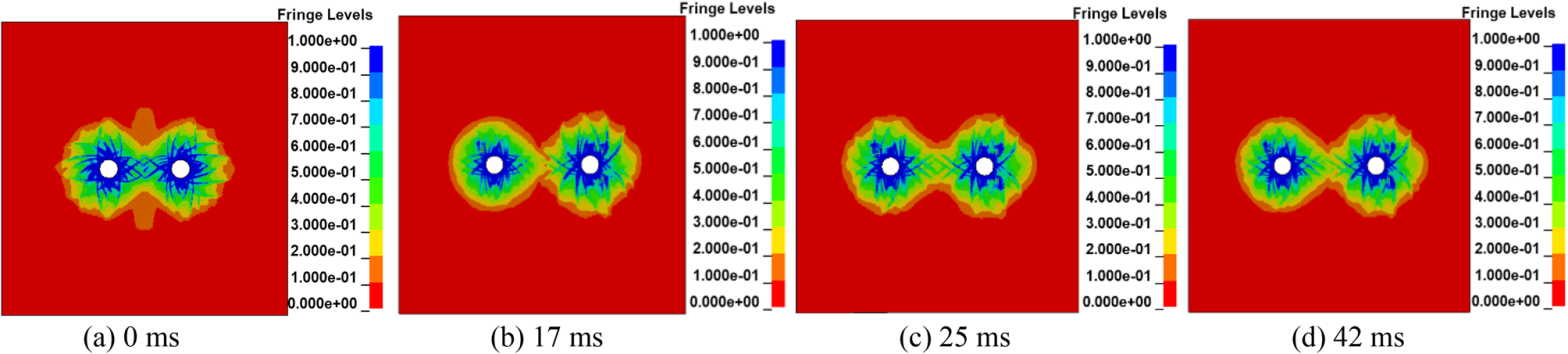
Cloud map of coal damage distribution at different delay times.

Figure 4 shows that a stress intersection will occur at a certain time after two blasting holes are detonated successively. Therefore, when the time interval of millisecond blasting is different, the distribution range of damage will be different. Taking Fig. 4a with a delay time of 0 ms as the control group, the crushing area of damage degree *D* in the range of 0.81 ~ 1 with delay times of 17 ms, 25 ms and 42 ms is basically the same, which is smaller than that of double hole simultaneous blasting. The results show that although delayed blasting cannot change the energy produced at the moment of explosion, the stress produced by blasting can be superimposed better by the time difference, and the action time on the medium can be prolonged to achieve the best blasting effect. It is not difficult to see from Fig. 4 that when the millisecond blasting time interval is 25 ms, most of the coal body damage degree *D* is concentrated in the range of 0.19 ~ 0.81. The results show that when the interval of millisecond blasting is 25 ms, the development of coal and rock fissures is better, the effect of stress waves on the medium is stronger, and the blasting effect is the best, which is helpful for gas drainage. Combined with the actual situation, the time interval of millisecond blasting is set as 25 ms.

### Influence analysis of the control hole on the blasting effect

**Figure 5:**
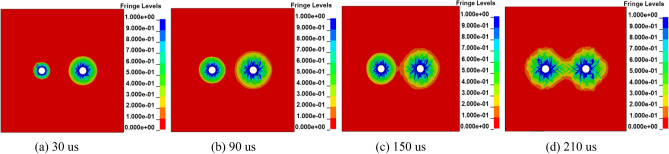
Damage evolution cloud map of coal without a control hole.

**Figure 6:**
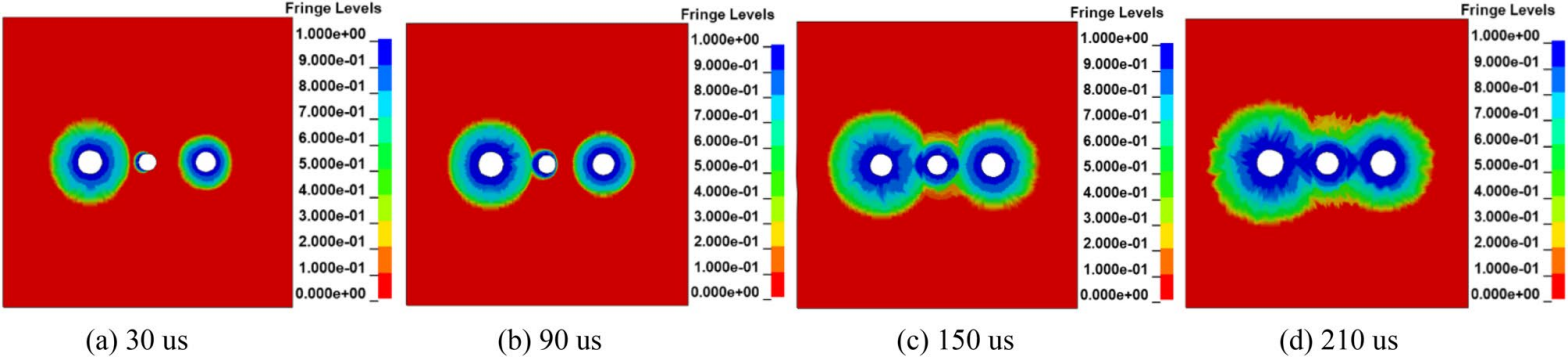
Damage evolution cloud map of coal with a control hole.

In actual blasting engineering, to improve the fracturing effect of coal and rock masses and reduce the cost, in addition to considering the above condition parameters, the role of the control hole is also particularly important. Based on the results of a previous study, the influence of the control hole is analysed by numerical simulation. Two groups of simulation experiments were established. The pore diameter was 113 mm, the distance between holes was 5.5 m, and the interval of millisecond blasting was 25 ms. The control holes were taken as the control variables. The simulation results are shown in Figs. 5 and 6.

It can be seen from Figs. 5 and 6 that after blasting, the effect range of stress produced by blasting is different with or without the influence of the control hole, and the damage range is wider due to the control hole. Especially, when blasting is carried out to 210 us, a large range of cracks will appear in the coal body under the two groups of experiments. However, the damage area with the control hole was larger than that without the control hole, and the stress was more concentrated. The results show that the control hole can not only control the direction of energy transfer but also improve the comprehensive effect of blasting so that the stress action time is longer and the coal fracture development is more sufficient.

## Industrial test on the permeability enhancement technology of deep hole presplitting blasting

### Overview of the test site

The test site is located at the 1312 transport roadway working face of coal seam #3 in the Sanyuan coal mine. The working face faces 1310 in the east, Shangqin village in the south, Changzhi South Station in the west, and 1309 goaf in the north, and G326 provincial highway is above the working face. According to the mining design of the 1312 working face in the No. 4 mining area of the Sanyuan coal mine, the mining ratio is 1:2. The maximum burial depth of the1312 working face in the No. 4 mining area of coal seam #3 is 900 m. The section of the working face is 18.2 m^2^. The effective advancing length of the working face along the strike is 1207.6 m. The length of incision 1 is 164.5 m. When the working face advances 249 m, the working face and open off cut 2 are combined, the working face is extended by 70 m, and the total length of the working face is 234.5 m.

The 1312 working face elevation of coal seam #3 is + 543 m ~  + 582 m, and the ground elevation is + 932 m ~  + 936 m. The gas content of coal seam #3 is 5.50–5.68 m^3^·t^-1^, the gas pressure is 0.36–0.39 MPa, and the attenuation coefficient of gas emission is 0.10966α*(d^-1^). The permeability coefficient of the coal seam is 0.0852 m^2^·(MPa^2^·d)—^1^, and the firmness coefficient of coal seam #3 is 0.816.

### Investigation of the radius of deep hole presplitting blasting

#### The setting of pumping holes

**Figure 7:**
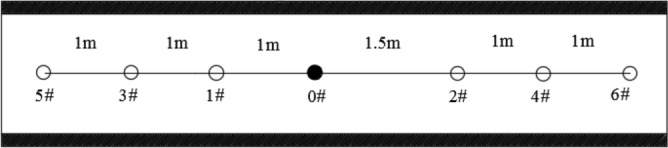
Drilling construction layout.

The effective extraction radius of the 1312 working face in coal seam #3 of the Sanyuan coal mine was determined by the relative pressure index method^37^. Combined with the actual situation of the site, a group of deep hole presplitting blasting effective radii was constructed in the 1312 working face to investigate the measuring points. Seven Φ113 bedding boreholes were constructed, and the sealing length was 12 m. The drilling construction layout is shown in Fig. 7.

#### Analysis of the effective extraction radius

**Figure 8:**
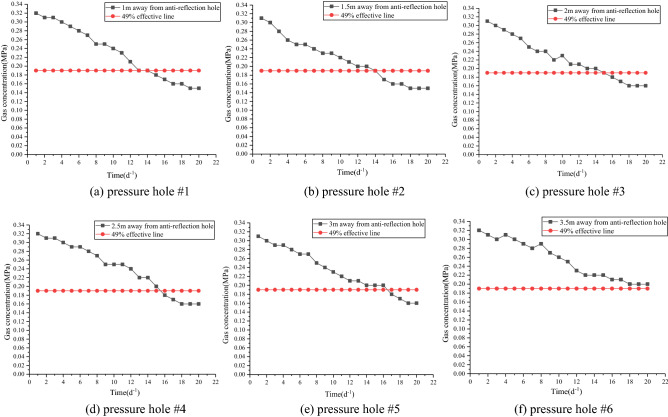
Gas pressure change curves of pressure taps.

The construction of an effective radius measurement borehole for drainage officially started on October 17, 2019. The pressure measuring hole was connected to the drainage system pipeline for drainage after 7 days of sealing. The gas pressure value in the borehole was recorded once a day. The measurement and recording time span was from October 24 to November 12, totalling 20 days. The gas pressure change curve of each pressure measuring hole is shown in Fig. 8.

Figure 8 shows that the pressures of five pressure measuring holes 1 m, 1.5 m, 2 m, 2.5 m and 3 m away from the permeability enhancement hole all decreased by more than 49% (to less than 0.19 MPa) during the 20 days of drainage, reaching the gas drainage index, while the gas pressure at the pressure tap 3.5 m away from the permeability enhancement hole did not drop below 0.19 MPa. In conclusion, when the extraction time is 20 days, the effective extraction radius is 3 m.

### Investigation of the permeability enhancement effect of deep hole presplitting blasting

#### Determination of the related parameters of blasting borehole

**Figure 9:**
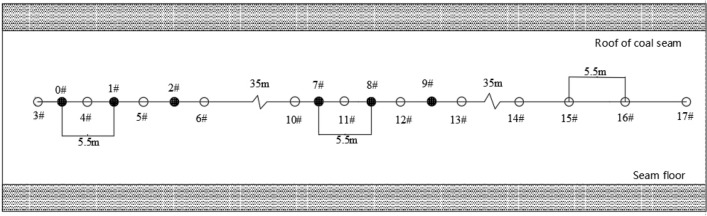
Schematic diagram of the drilling construction at the test site.

The test was carried out at a 400 m distance from the open cutting hole of the 1312 working face of the transportation entry of coal seam #3 in the Sanyuan coal mine. Three blasting holes were constructed. The spacing of the holes was 5.5 m, the distance between the observation hole and the blasting hole was 2.25 m, and the delay time was set at 25 ms. The numbers of drill holes were #7, #8 and #9, and the detonation started from hole #7 successively. To avoid the interference of blasting shock waves, four natural drainage holes #14, #15, #16, and #17 were constructed 35 m away from hole #9. The layout of the drilling is shown in Fig. 9. After the completion of borehole sealing, the drainage pipeline, drainage concentration and drainage flow detection equipment should be installed to conduct gas drainage for 30 days. The permeability enhancement effect of deep hole presplitting blasting was determined by investigating the variation in natural gas emissions, coal seam permeability coefficient and presplitting blasting cracking effect.

#### Characteristics of natural gas emissions from boreholes

**Figure 10:**
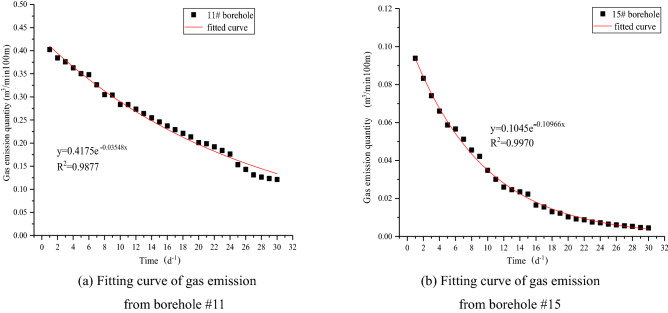
Characteristics of natural gas emissions from 100 boreholes.

Observation hole #11 and common extraction hole #15 of the 1312 working face of coal seam #3 were selected to measure the natural discharge flow rate of the borehole. Through the integration of monitoring data, the gas flow attenuation curves of boreholes #11 and #15 were fitted, as shown in Table 3 and Fig. 10.

**Table 3:** 1312 Results of natural gas gushing from a 100-m borehole of the working face.

Number of drill	Gushing law *q*_t_ = *q*_o_e^-*αt*^	Initial gas emission intensity m^3^·(min·100 m)^−1^	Attenuation coefficient of natural gas flow in the borehole (d^−1^)
#11	*q*_t_ = 0.4175e^−0.03548*t*^	0.4175	0.03548
#15	*q*_t_ = 0.1045e^−0.10966*t*^	0.1045	0.10966

According to the law of natural gas emission from boreholes #11 and #15 in Table 3, after presplitting blasting, the attenuation coefficient of gas flow in the borehole decreases from *α*_15_ = 0.10966 d^-1^ for hole #15 to *α*_11_ = 0.03548 d^-1^ for hole #11, that is, *α*_11_ = 0.3235*α*_15_. The results show that the attenuation intensity of gas emission decreased by 67.65% after the implementation of deep hole presplitting blasting on working face 1312. This means that the permeability enhancement of presplit blasting changes the pore structure of the coal, which is conducive to continuous gas extraction.

After measuring the natural discharge flow of observation hole #11 and ordinary extraction hole #15, these two holes should be blocked to prevent them from being in an open discharge state to avoid affecting the blasting hole and surrounding extraction hole.

#### Permeability coefficient change test of the coal seam

**Figure 11:**
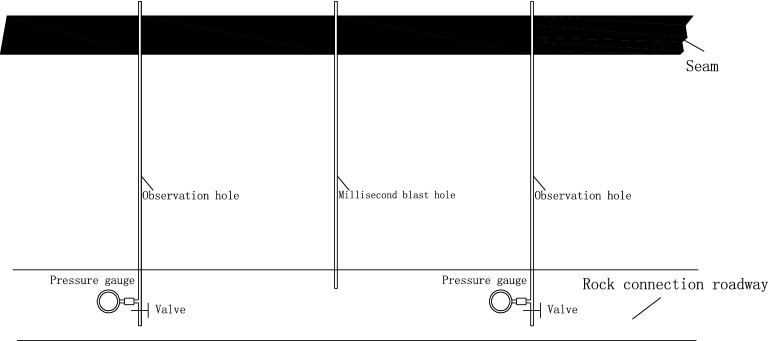
Schematic diagram of the drilling of coal seam permeability coefficient construction at the test site.

The arrangement of boreholes for measuring the permeability coefficient of the coal seam is shown in Fig. 11, and the parameters are shown in Table 4.

**Table 4:** Drilling parameters.

Name	Angle with roadway	Elevation	Drilling depth	Visible coal length	Pressure	Distance from the blasting hole
Value 1	90	+ 40	25	7.5	0.24	2.25
Value 2	90	+ 40	25	7.5	0.26	2.25
Unit	°	°	m	m	MPa	m

According to the calculation formula of the permeability coefficient^38^, after deep hole presplitting blasting, the permeability of the original coal seam is increased from 0.0852 m^2^·(MPa^2^·d)^−1^ to 2.565 m^2^·(MPa^2^·d)^−1^ ~ 3.278 m^2^·(MPa^2^·d)^−1^, which is an increase of more than 30 times, and the permeability of the coal seam is greatly improved.

### Comparison of the gas extraction effect before and after blasting

The most direct purpose of deep hole presplitting blasting is to produce a large number of cracks in the coal body to improve the permeability of the coal mass. Therefore, the gas drainage effect before and after blasting is judged by monitoring and recording the changes in gas concentration, gas purity and gas mixture parameters in the gas drainage pipeline and drawing a curve diagram. The observation time is 40 days.

**Figure 12:**
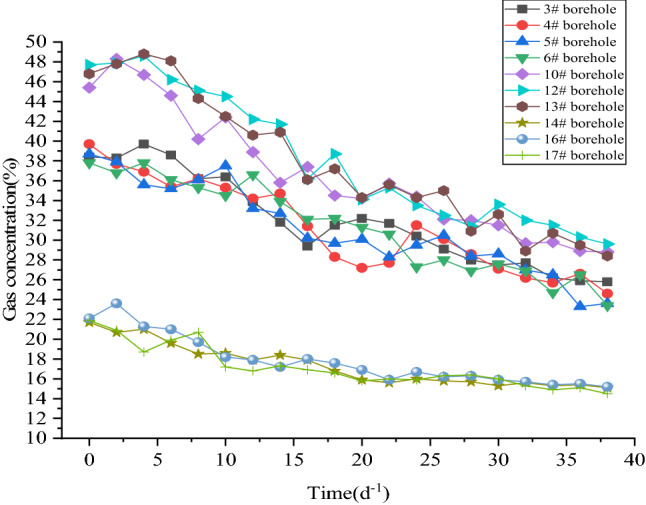
Comparison of the gas concentration in boreholes.

It can be seen from Fig. 12 that the gas concentration of ordinary extraction holes fluctuates between 15.0 and 23.6%, and the average gas concentration is 17.44%. The gas concentration in the observation hole after blasting varied between 28.4 and 48.9%, and the average gas concentration was 37.60%. After deep hole presplitting blasting, the average gas extraction concentration increased by 2.16 times. The gas drainage period is longer, and the drainage effect is better.

**Figure 13:**
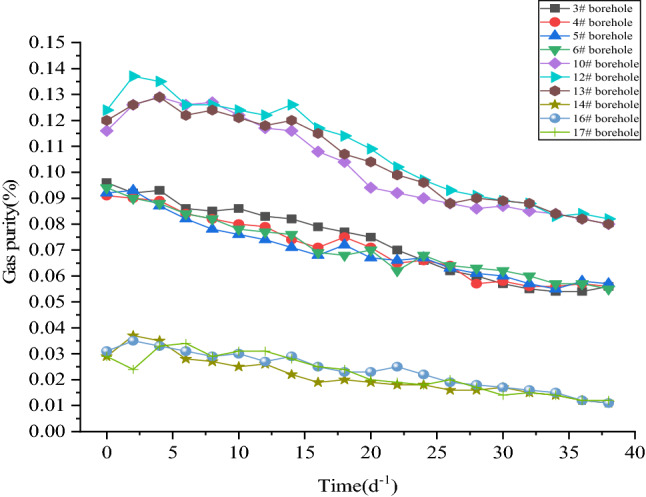
Single hole gas purity quantity comparison.

**Figure 14:**
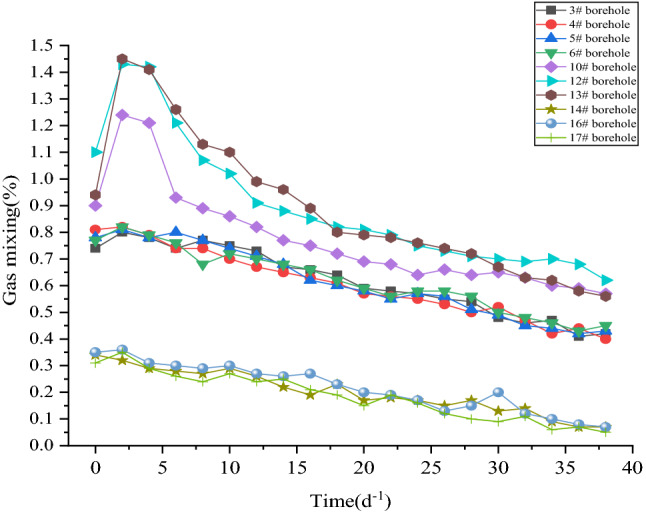
Single hole gas mixing quantity comparison.

It can be seen from Figs. 13 and 14 that the gas purity range of normal extraction holes #14, #16, and #17 fluctuates between 0.011 and 0.037 m^3^·min^-1^. The average borehole purity is 0.0223 m^3^·min^-1^. The gas mixing volume of a single hole fluctuates between 0.005 and 0.35 m^3^·min^-1^. The average borehole mixture is 0.1975 m^3^·min^-1^. After the implementation of deep hole presplitting blasting permeability increasing technology, the single hole gas purity of observation boreholes #10, #12, and #13 varies from 0.080 to 0.137 m^3^·min^-1^. The average single hole gas purity is 0.1055 m^3^·min^-1^, and the average single hole purity is increased by 4.73 times. The gas mixing quantity of a single hole in observation boreholes #10, #12, and #13 varies from 0.56 to 1.45 m^3^·min^-1^, and the average gas mixing quantity of a single hole is 0.8425 m^3^·min^-1^, which is an increase of 4.27 times.

In summary, after the implementation of deep hole presplitting blasting, the cracks in the coal body are extended, which greatly improves the amount of gas extraction in the coal seam. The results show that new irreversible cracks are produced by presplitting blasting. This shows that deep hole presplitting blasting technology is effective in promoting gas drainage in the Sanyuan coal mine.

## Conclusion

1. The damage degree of coal rock blasting is quantitatively evaluated by using the value range of the damage variable *D*. According to the actual field test parameters of coal seam #3 in the Sanyuan coal mine, when *D*_lim_ is equal to 0.81 ~ 1.0, it is a coal rock crushing area. When *D*_lim_ is equal to 0.19 ~ 0.81, it is a fracture zone. When *D*_lim_ is equal to 0 ~ 0.19, it is a coal rock disturbance area.
2. The blasting model is established under different blasting parameters. The blasting parameters are optimized using ANSYS/LS-DYNA software. The blasting cracking effect is analysed by observing the cloud chart of the coal damage distribution. The blasting impact radius is determined to be approximately 3.1 m by taking the distribution characteristics of the coal blasting damage area as the evaluation index. The simulation results of double blasting holes show that when the blasting hole diameter is 113 mm and the hole spacing is 5.5 m, controlling the delayed blasting time at 25 ms improves the effect of permeability enhancement technology.
3. The measured effective radius of gas extraction is 3.0 m by the relative pressure index method, which is basically consistent with the numerical simulation results. According to the numerical simulation results, the best blasting parameters are tested in the field. The results are as follows: the attenuation coefficient of borehole gas flow decreases from 0.1096 d^−1^ to 0.03548 d^−1^. The attenuation intensity of gas emission from the borehole decreased by 67.65%. The air permeability of the original coal seam increased from 0.0852 m^2^·(MPa2·d)^−1^ to 2.565 m^2^·(MPa2·d)^−1^ ~ 3.278 m^2^·(MPa2·d)^−1^, increasing by more than 30 times. The gas extraction concentration increased by 2.16 times. The single-hole purity and mixing quantity increased by 4.73 times and 4.27 times, respectively. The extraction effect is significantly improved after deep hole presplitting blasting.
4. In this paper, the single hole diameter, blast hole spacing, delayed blasting time, etc. are selected as the optimization parameters. However, the influence of gas pressure, ground stress and coal body firmness coefficient on blasting has not been considered in detail and has certain limitations. In follow-up research, we can consider analysing the three parameters and conducting more in-depth research on the selection of blasting optimization parameters.
